# Analysis of predicted proteasomal cleavages in the methyltransferase domain from JEV

**DOI:** 10.6026/97320630016223

**Published:** 2020-03-31

**Authors:** Sarah Afaq, Arshi Malik, Md.Salman Akhtar, Afaf S Alwabli, Dhafer A Alzahrani, Habeeb M Al-Solami, Othman Alzahrani, Qamre Alam, Mohammad Azhar Kamal, Aala A Abulfaraj, Mohammad Tarique

**Affiliations:** 1 Department of Clinical Biochemistry, College of Medicine, King Khalid University, Abha, Kingdom of Saudi Arabia; 2 Department of Clinical Biochemistry, College of Medicine, King Khalid University, Abha, Kingdom of Saudi Arabia; 3 Department of Basic Medical Sciences, Faculty of Applied Medical Sciences, Al-Baha University, Al-Baha, Kingdom of Saudi Arabia; 4 Department of Biological Sciences, Faculty of Science, King Abdulaziz University, Jeddah 21589, Kingdom of Saudi Arabia; 5 Department of Biological Sciences, Faculty of Science, King Abdulaziz University, Jeddah 21589, Kingdom of Saudi Arabia; 6 Department of Biological Sciences, Faculty of Science, King Abdulaziz University, Jeddah 21589, Kingdom of Saudi Arabia; 7 Department of Biology, Faculty of Science, University of Tabuk, Tabuk, Kingdom of Saudi Arabia; 8 Medical Genomics Research Department, King Abdullah International Medical Research Center, King Saud bin Abdulaziz University for Health Sciences, Ministry of National Guard Health Affairs, Riyadh, Saudi Arabia; 9 Department of Biochemistry, Faculty of Science, University of Jeddah, Jeddah, Saudi Arabia; 10 University of Jeddah Center for Science and Medical Research (UJC-SMR), Jeddah, Saudi Arabia; 11 Department of Biology, Science and Arts-Rabigh Campus, King Abdulaziz University, Jeddah, Saudi Arabia; 12 Center for Interdisciplinary Research in Basic Sciences, Jamia Millia Islamia, Jamia Nagar, New Delhi-110025, India

**Keywords:** Japanese encephalitis (JEV), infection, methyltransferase, proteasome, cleavage

## Abstract

The methyltransferase (MTase, a 265 amino acid residues long region at the N-terminal end of the viral nonfunctional supermolecule NS5 domain) is key for viral replication in Japanese Encephalitis Virus (JEV). Sequence to structure to functional information with adequate knowledge on MTase from JEV is currently limited. Therefore, it is of interest to document a report on the comprehensive analysis of predicted proteasomal cleavage data in the methyltransferase domain from JEV. This data is relevant in the design and development of vaccine and other therapeutic candidates for further consideration.

## Background

Japanese Encephalitis (JEV) is an infection, which belongs to the family of Flaviviridae. It is the cause for Viral Encephalitis worldwide with 50,000 cases every year and 15,000 deaths [1].The genome of the Flavivirus is a reclusive stranded RNA having a positive end [2,3].The genome of the Flavivirus encodes a polyprotein with proteases divided into 7 nonstructural proteins such as NS1, NS2A, NS2B, NS3, NS4A, NS4B and NS5 and NS3 along with envelope proteins [4].The non-structural 5 protein contains a N-terminal region named MTase (methyltransferase) and a C-terminal region named RdRp (RNA dependent RNA polymerase) [5-7].The link between proteasomal cleavage and cell mediated immune response well documented [8].Several methods to study proteasome cleavage are available [9-12].It is of interest to document a report on the comprehensive analysis of predicted proteasomal cleavage data in the methyltransferase domain from JEV towards the development of suitable therapeutics against the virus.

## Methodology

### Conserved domain:

The MTase domain sequence was downloaded from NCBI Genome database is 265 amino acids. We analyzed the sequence using BLASTP for identifying homologs. The sequence was further analyzed using Prosite, SMART, PANTHER, Pfam and InterProScan for functional annotation of the MTase sequence [13-15]. The MTase domain consisted of a mRNA cap binding region containing 13 amino acids along with a carbonyl oxygen region containing 16 amino acids and a S-adenosyl-L-methionine containing 56 amino acids. These are necessary for the activity of 2'-O-methyltransferase made of 61 amino acids. Moreover, the ends of MTase comprises of some coils, helices and strands.

### Sequence logo analysis:

We analyzed the MTase sequence using a Sequence Logo Generator as described elsewhere [16,17] to identify patterns as logos in the protein.

### Secondary structures:

We used PSIPRED (http://bioinf.cs.ucl.ac.uk/psipred/) to assign secondary structures in the MTase domain sequence.

### Epitope prediction:

We used the Penchant scale (http://tools.immuneepitope.org) for assign epitope properties in the MTase domain under analysis [18].

### B-cell epitope prediction:

Conformational B-cell epitopes were predicted using the tool available at http://tools.immuneepitope.org and as described elsewhere [18-24].

### Proteasomal cleavage prediction

Proteosomal cleavage prediction was completed using NetCTL [25], NetChop and NetCTLpan [26].

## Results and Discussion:

**Figure 1:**
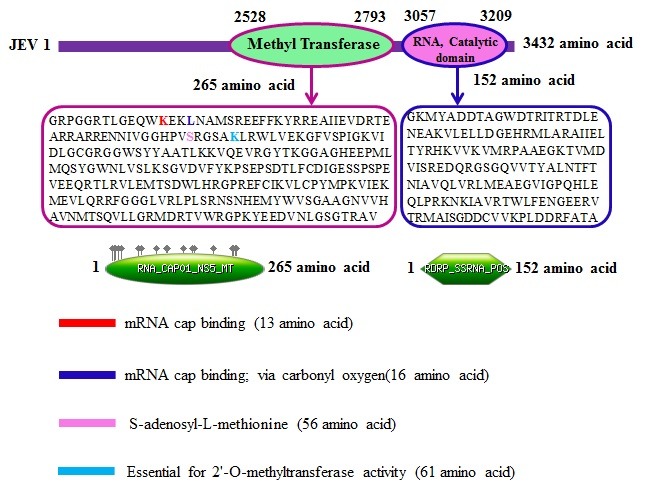
Domain organization in the JEV proteome is shown. It consists of two domains (MTase and RdRP) as shown.

**Figure 2:**
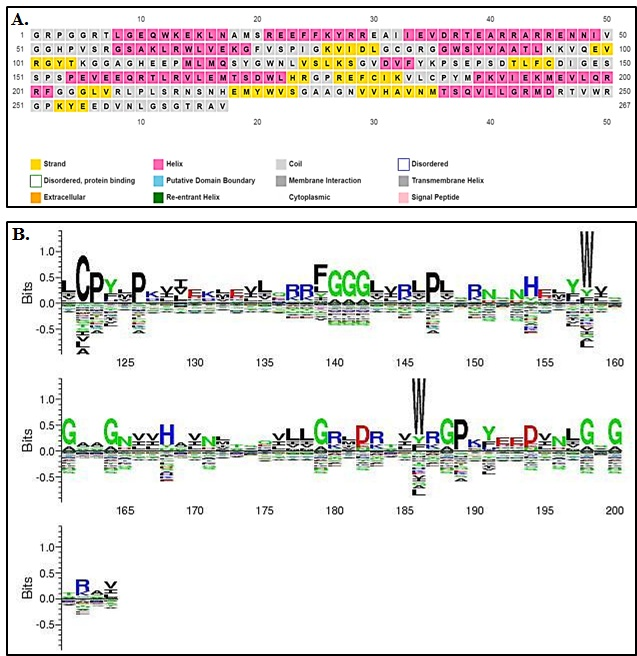
The protein sequence of the MTase domain from JEV is shown with (A) secondary structures and (B) conserved domains.

**Figure 3:**
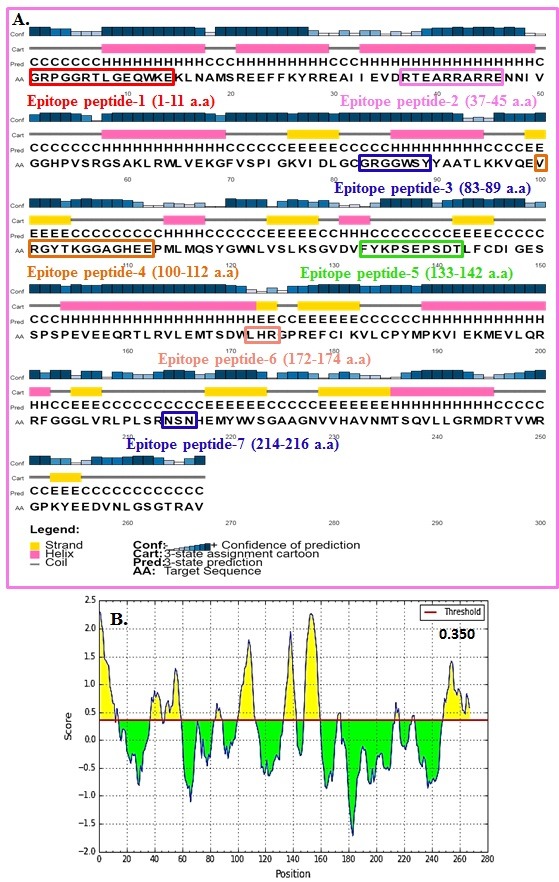
MTase with secondary structures (A) and conformational epitopes (B) is shown.

**Figure 4:**
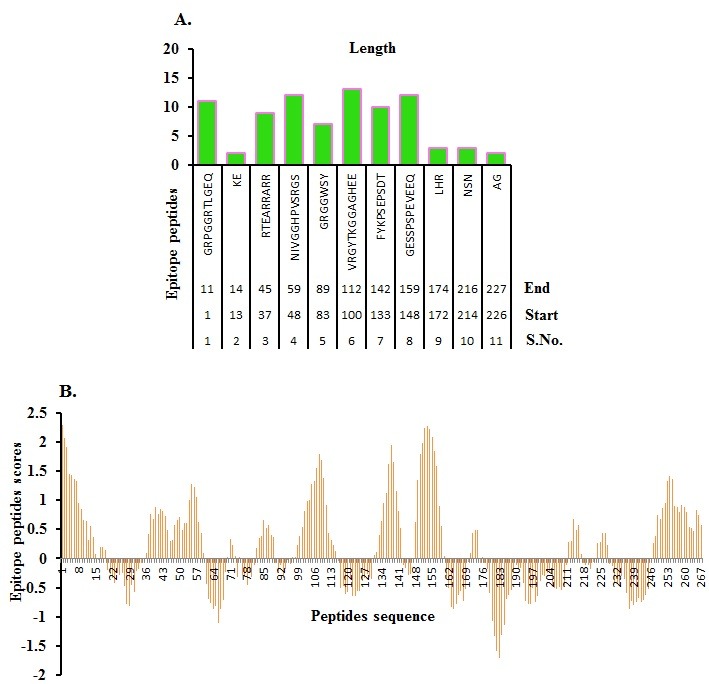
Epitopes in MTase with (A) peptides with position and (B) predicted antigen score

**Figure 5:**
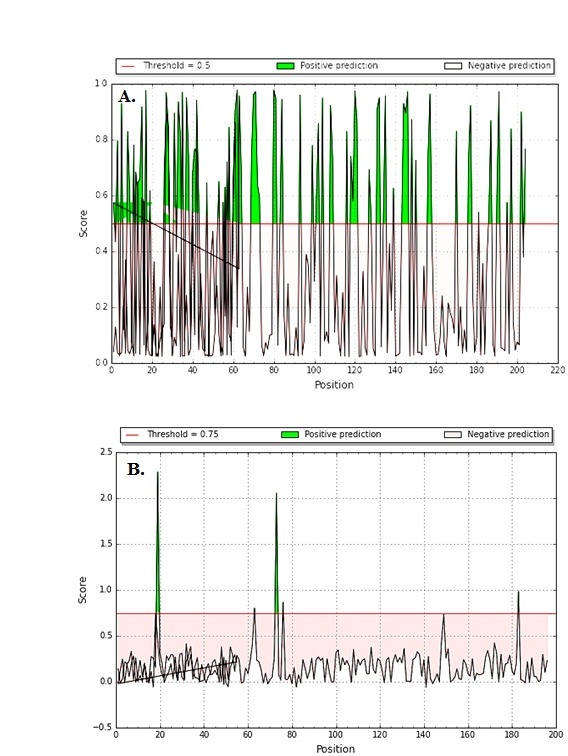
Predicted cleavage sites in MTase with (A) score and expanded region with score (B)

The methyltransferase (MTase, a 265 amino acid residues long region at the N-terminal end of the viral nonfunctional supermolecule NS5 domain) is key for viral replication in Japanese Encephalitis Virus (JEV). Sequence to structure to functional information with adequate knowledge on MTase from JEV is currently limited. Therefore, it is of interest to document a report on the comprehensive analysis of predicted proteasomal cleavage data in the methyltransferase domain from JEV. Domain organization in the JEV proteome is shown in (Figure 1). It consists of two domains (MTase and RdRP). The protein sequence of the MTase domain from JEV is shown with secondary structures and conserved domains in (Figure 2) as described elsewhere [27]. MTase with secondary structures and conformational epitopes is shown in (Figure 3) as described elsewhere [28-30]. Epitopes (antigenic regions) in MTase with short peptides with residue position and predicted antigen score is given in (Figure 4) as described elsewhere [28-30]. Data on predicted cleavage sites in MTase with score and expanded region with score is given in (Figure 5) as described elsewhere [31-34]. Thus, a comprehensive analysis of the MTase domain in JEV is highly important for further understanding of the sequence to structure to functional analysis of the protein. This data is relevant in the design and development of vaccine and other relevant therapeutic candidates for further consideration.

## Conclusions:

We report a preliminary analysis of predicted proteasomal cleavage data in the methyltransferase domain from JEV. This data is relevant in the design and development of vaccine and other therapeutic candidates for further consideration.
